# Development of Mannose-Modified Carboxylated Curdlan-Coated Liposomes for Antigen Presenting Cell Targeted Antigen Delivery

**DOI:** 10.3390/pharmaceutics12080754

**Published:** 2020-08-11

**Authors:** Eiji Yuba, Yoshiki Fukaya, Shin Yanagihara, Nozomi Kasho, Atsushi Harada

**Affiliations:** Department of Applied Chemistry, Graduate School of Engineering, Osaka Prefecture University, 1–1 Gakuen–cho, Naka–ku, Sakai, Osaka 5998531, Japan; sxb02120@gmail.com (Y.F.); syb02130@edu.osakafu-u.ac.jp (S.Y.); szb02036@edu.osakafu-u.ac.jp (N.K.)

**Keywords:** liposome, mannose, polysaccharide, macrophage, cross-presentation, cellular immunity

## Abstract

Specific delivery to antigen presenting cells (APC) and precise control of the intracellular fate of antigens are crucial to induce cellular immunity that directly and specifically attacks cancer cells. We previously achieved cytoplasmic delivery of antigen and activation of APC using carboxylated curdlan-modified liposomes, which led to the induction of cellular immunity in vivo. APCs express mannose receptors on their surface to recognize pathogen specifically and promote cross-presentation of antigen. In this study, mannose-residue was additionally introduced to carboxylated curdlan as a targeting moiety to APC for further improvement of polysaccharide-based antigen carriers. Mannose-modified curdlan derivatives were synthesized by the condensation between amino group-introduced mannose and carboxy group in pH-sensitive curdlan. Mannose residue-introduced carboxylated curdlan-modified liposomes showed higher pH-sensitivity than that of liposomes modified with conventional carboxylated curdlan. The introduction of mannose-residue to the liposomes induced aggregation in the presence of Concanavalin A, indicating that mannose residues were presented onto liposome surface. Mannose residue-introduced carboxylated curdlan-modified liposomes exhibited high and selective cellular association to APC. Furthermore, mannose residue-introduced carboxylated curdlan-modified liposomes promoted cross-presentation of antigen and induced strong antitumor effects on tumor-bearing mice. Therefore, these liposomes are promising as APC-specific antigen delivery systems for the induction of antigen-specific cellular immunity.

## 1. Introduction

Cancer immunotherapy has been gained much attention as one of cancer treatment modalities since the success of immune checkpoint inhibitors in a clinic [1,2]. Immune checkpoint inhibitors show therapeutic effects in cancer via interfering immunosuppression signals, such as CTLA-4, PD-1, PD-L1, and so on [3,4,5,6]. However, currently available immune checkpoint inhibitors exhibit therapeutic effects in only 20–30% of cancer patients probably because of other immunosuppression mechanisms dominant in most patients [3,7]. For example, CD47-mediated macrophage suppression is regarded as a potent candidate for other immunosuppression mechanisms and clinical studies using anti-CD47 antibody are ongoing [8,9]. On the other hands, after cancel of immunosuppression in tumor microenvironments by these immunomodulatory molecules, cell-based immunity (cellular immunity) plays a crucial role to regress tumor burden directly. Therefore, the activation strategy of tumor-specific cellular immunity is another issue to achieve efficient cancer immunotherapy.

For the induction of cancer-specific immunity, cancer antigens should be processed in antigen presenting cells (APCs), such as dendritic cells, macrophages, and B cells [10,11,12]. The intracellular fate of the antigen determines the induction mode of immune responses. When antigens are degraded in endo/lysosomes and are carried onto major histocompatibility complex (MHC) class II molecules, helper T lymphocyte-mediated humoral immunity is induced. When antigens are introduced into cytosol, processed in proteasomes and carried onto MHC class I molecules, cytotoxic T lymphocyte (CTL)-based cellular immunity is induced. Therefore, precise control of the intracellular delivery process of cancer antigens is required to activate CTL-based cellular immunity.

Presentation of exogenous antigen onto MHC I molecules is also called as “cross-presentation” [13,14,15]. So far, various types of antigen delivery carriers that can induce cross-presentation have been reported [16,17,18,19,20]. Major strategy for cross-presentation is cytoplasmic delivery of antigen via fusion with plasma membrane or endosomal membrane using fusogenic protein/peptide-introduced nanocarriers or synthetic molecule-modified nanocarriers that can promote endosomal escape of antigen [21,22,23]. We have also reported cytoplasmic delivery system of antigen using pH-responsive polyglycidols or polysaccharides [24,25,26]. These polymer-modified liposomes containing antigenic proteins or peptides could deliver antigens into cytosol of APC via fusion with endosomal membrane responding to weakly acidic pH in endosomes [25]. In addition, these polymer-modified liposomes could achieve cross-presentation in dendritic cells and the induction of antigen-specific cellular immunity in vivo [25,27]. Furthermore, the use of bioactive polysaccharide as a backbone of pH-responsive polymer improved immunity-inducing functions [28]. Curdlan, a linear β-glucan existing in bacterial cell wall, is known to activate immune cells via its intrinsic adjuvant property derived from recognition by β-glucan receptor (Dectin-1). Indeed, pH-sensitive group-introduced curdlan-modified liposomes exhibited efficient cytoplasmic delivery performance and activation of APCs [28]. However, the antitumor effect by curdlan derivative-modified liposomes was not enough to eradicate tumor burden completely. Therefore, further improvements are required for these antigen delivery systems.

Most of macrophages and dendritic cells express mannose receptors to recognize high mannose polysaccharide-bearing bacteria to initiate immune responses against these pathogens [29,30]. Therefore, introduction of mannose residues onto antigen delivery carriers is expected to promote recognition by APC. Indeed, oligomannose-bearing lipid-incorporated liposomes, mannose-bearing alginate-based nanoparticles, and sugar moieties-introduced poly(glycidyl methacrylate)-based nanoaggregates were fabricated towards APC-specific antigen delivery [31,32,33,34]. In addition to APC-specific delivery performance, mannose receptor-mediated antigen uptake by dendritic cells is known to promote cross-presentation via weakly acidic intracellular compartments, which is called as “vacuolar pathway” [13,14]. Hence, mannose modification is regarded as a reasonable strategy to improve the antigen delivery performance of antigen carriers.

**Figure 1:**
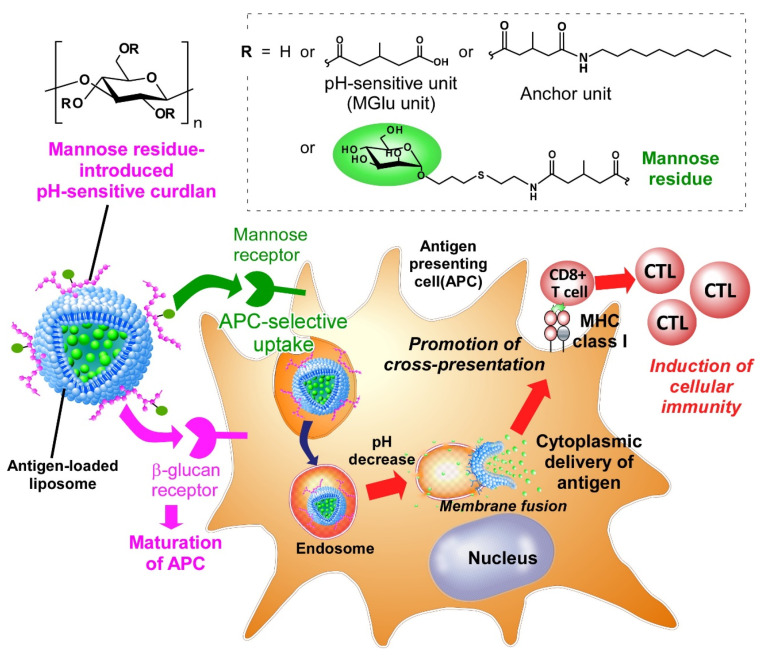
Design of mannose residue-introduced pH-responsive curdlan-modified liposomes for targeting to antigen presenting cell and cytoplasmic delivery of antigen towards induction of cross-presentation and cellular immunity.

In this study, the feasibility of mannose modification to pH-responsive curdlan-modified liposomes was investigated. Curdlan derivatives having 3-methylglutaric acid ester units (MGlu units) as a pH-sensitive unit and decylamidated units as an anchor unit to liposomal membrane (MGlu-Curd-A) were further functionalized by mannose residues (Figure 1). Obtained curdlan derivatives (MGlu-Curd-A-Man) were modified onto antigen-loaded liposomes. These liposomes are expected to promote APC-specific uptake via recognition by mannose receptors, activate APC via recognition by curdlan receptor (Dectin-1) and induce cytoplasmic delivery of antigen, which leads to cross-presentation and induction of antigen-specific cellular immunity (Figure 1). Here, the synthesis of mannose residue-introduced carboxylated curdlans, pH-sensitivity, selectivity to APC, cross-presentation efficacy, and antitumor effects of curdlan derivative-modified liposomes were investigated.

## 2. Materials and Methods

### 2.1. Materials

Egg yolk phosphatidylcholine (EYPC) was kindly donated by NOF Co. (Tokyo, Japan). Allyl-α-D-mannopyranoside, cysteamine hydrochloride, 3-methylglutaric anhydride, Curdlan from *Alcaligenes faecalis*, ovalbumin (OVA), bovine serum albumin (BSA), *p*-xylene-bis-pyridinium bromide (DPX) and calcein were purchased from Sigma (St. Louis, MO, USA). Triton X-100, 1-Aminodecane, and pyranine were obtained from Tokyo Chemical Industries Ltd. (Tokyo, Japan). Concanavalin A and 4-(4,6-Dimethoxy-1,3,5-triazin-2-yl)-4-methyl morpholinium chloride (DMT-MM) were obtained from Wako Pure Chemical Industries Ltd. (Osaka, Japan), while 1,1′-Dioctadecyl-3,3,3′,3′-tetramethylindocarbocyanine perchlorate (DiI) was from Life Technologies.

### 2.2. Synthesis of Mannose-Modified Curdlan Derivatives

**Scheme 1:**
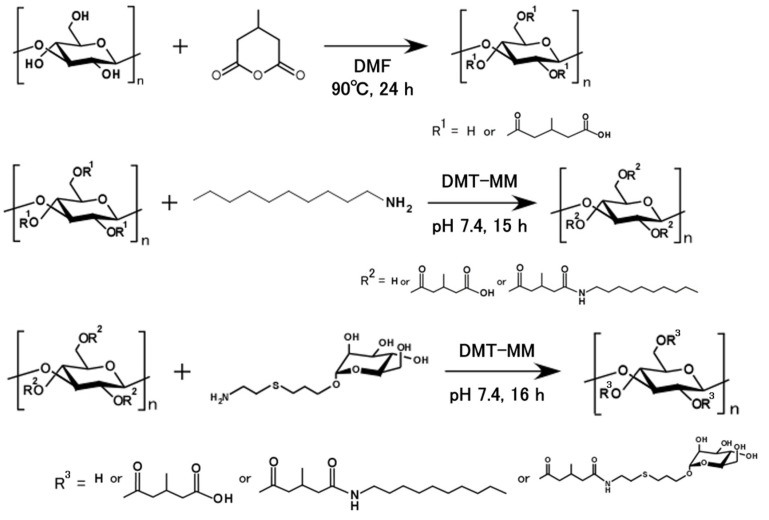
Synthetic route of MGlu-Curd-A-Man.

Briefly, 3-methylglutarylated Curdlan having an anchor moiety (MGlu-Curd-A, Man0) were synthesized via the esterification of curdlan using 3-methylglutaric anhydride and subsequent condensation with 1-aminodecane according to the previous literature [28] (Scheme 1). Mannose residue (6-amino-4-thiahexyl α-D-mannopyranoside) was synthesized via thiol-ene reaction of allyl-α-D-mannopyranoside and cysteamine hydrochloride as shown in Supplementary Scheme S1 [35]. A given amount of 6-amino-4-thiahexyl α-D-mannopyranoside was reacted with carboxy groups of MGlu-Curd-A using DMT-MM at room temperature for 16 h with stirring (Scheme 1 and Supplementary Table S1). Obtained polymers were purified by dialysis against water. The product was recovered by freeze-drying and characterized using ^1^H NMR (Supplementary Figure S2). ^1^H NMR (400 MHz, D_2_O + NaOD) for hydrolyzed MGlu-Curd-A-Man: δ (ppm) 3.96–3.38 (br, glucose 2*H*, 3*H*, 4*H*, 5*H*, 6*H*, mannose 2*H*, 3*H*, 4*H*, 5*H*, 6*H*, Man-O-C*H*_2_-CH_2_-S-CH_2_-C*H*_2_-NH-), 2.77–2.65 (br, Man-O-CH_2_-C*H*_2_-S-C*H*_2_-CH_2_-NH-), 2.30–2.16 (br, -CO-C*H*_2-_C*H*(CH_3_)-C*H*_2_-), 2.08–1.94 (br, -CO-C*H*_2_-CH(CH_3_)-C*H*_2_-), 1.56–1.23 (br, decyl), 0.98–0.86 (m, -CO-CH_2-_CH(C*H*_3_)-CH_2_-).

### 2.3. Preparation of Curdlan Derivative-Modified Liposomes

A dry, thin membrane composed of a mixture of EYPC and curdlan derivatives (lipid/polymer = 7/3, *w*/*w*) was dispersed in PBS containing OVA (4 mg/mL) by a brief sonication, and the liposome suspension was further hydrated by freezing and thawing and was extruded through a polycarbonate membrane with a pore size of 200 nm. The liposome suspension was purified with Sepharose 4B column using PBS as an eluent.

### 2.4. Characterization of Curdlan Derivative-Modified Liposomes

Diameters of the liposomes (0.2 mM of lipid concentration) in PBS and zeta potentials of the liposomes (0.1 mM of lipid concentration) in 0.1 mM phosphate aqueous solution (pH 7.4) at 25 °C were measured using a Zetasizer Nano ZS (Malvern Instruments Ltd., Worcestershire, UK). Data were obtained as an average of more than three measurements on different samples. The concentrations of lipid and OVA in liposome suspension were measured using phospholipid C test-Wako and Coomassie Protein Assay Reagent, respectively.

Pyranine-loaded liposomes were prepared as described above except that mixture of curdlan derivative and EYPC was dispersed in aqueous 35 mM pyranine, 50 mM DPX, and 25 mM phosphate solution (pH 7.4). Pyranine-loaded liposomes (lipid concentration: 2.0 × 10^−5^ M) were added to PBS the pH of which was adjusted by the addition of HCl at 37 °C and fluorescence intensity at 512 nm of the mixed suspension was followed with excitation at 416 nm using a spectrofluorometer (FP-6500, Jasco, Tokyo, Japan). The percent release of pyranine from liposomes was defied as:

(1) $$\mathrm{Release}\left(\%\right)=\left(F_{t}-F_{i}\right)/\left(F_{f}-F_{i}\right)\times 100,$$

where *F*_i_ and *F*_t_ mean the initial and intermediary fluorescence intensities of the liposome suspension, respectively. *F*_f_ is the fluorescent intensity of the liposome suspension after the addition of TritonX-100 (final concentration: 0.1%). TritonX-100 was added after 30 min because pyranine release profile reached to the plateau in almost all measurements. Solution pH after measurements was measured and plotted in each panel. For evaluation of interaction between liposomes, pyranine-loaded unmodified liposomes (lipid concentration: 1.0 × 10^−5^ M) and OVA-loaded liposomes modified with curdlan derivatives (lipid concentration: 1.0 × 10^−5^ M) were mixed in PBS of varying pH at 37 °C. The percent release of pyranine from the liposomes was measured as described above.

### 2.5. Interaction of Curdlan Derivative-Modified Liposomes with Lectin

Turbidity change at 360 nm of liposome suspension (0.1 mM) in the presence of Concanavalin A (1 mg/mL) was measured using UV-VIS spectrometer (V-560, Jasco, Tokyo, Japan) to detect aggregation of liposomes via interaction with lectin. As a control experiment, BSA (1 mg/mL) was used instead of Concanavalin A. An excess amount (>100 equivalents) of free mannose was also added to the mixture of liposome and Concanavalin A to interrupt the interaction with lectin.

### 2.6. Cellular Association of Liposomes

Liposomes containing DiI were prepared as described above except that a mixture of curdlan derivatives and EYPC containing DiI (0.1 mol%) was dispersed in PBS containing OVA. RAW264.7 cells, a murine macrophage cell line, or NIH3T3 cells, a murine fibroblast (7.5 × 10^4^ cells) cultured for 2 days in a 24-well plate were washed twice with PBS and then incubated in medium with or without serum (0.25 mL). DiI-labeled liposomes (0.2 mM lipid concentration, 0.25 mL) were added gently to the cells and incubated for 4 h at 37 °C. After incubation, the cells were washed with PBS three times. Fluorescence intensity of these cells was determined via a flow cytometric analysis (CytoFlex, Beckman Coulter, Inc., Brea, CA, USA). Relative fluorescence intensity for each liposome was calculated using fluorescence intensity for the cells without treatment.

### 2.7. Confocal Laser Scanning Microscpy

DiI-labeled liposomes containing calcein were prepared as described above except that a mixture of curdlan derivatives and EYPC containing DiI (0.1 mol%) was dispersed in 63 mM calcein solution. RAW264.7 cells (1.5 × 10^5^ cells) cultured for 2 days in a glass-bottom dish were washed twice with PBS and then incubated in a medium with serum (0.5 mL). DiI-labeled liposomes (0.2 mM lipid concentration, 0.5 mL) were added gently to the cells and incubated for 4 h at 37 °C. After incubation, the cells were washed with PBS three times and observed using confocal laser scanning microscopy LSM5 EXCITER (Carl Zeiss, Oberkochen, Germany).

### 2.8. Evaluation of Antigen Presentation

RAW264.7 cells (1.5 × 10^5^ cells) cultured for 2 days in a six-well plate were washed with PBS twice and then incubated in culture medium. OVA-loaded liposomes (final lipid concentration: 0.1 mM) or OVA epitope peptide (SIINFEKL) were added gently to the cells, followed by incubation for 24 h at 37 °C. After incubation, the cells were washed with PBS three times and the presentation of OVA peptide on the cell surface was detected as follows: 3 × 10^5^ of cells were re-suspended in the staining buffer (PBS containing 10% BSA and 1% NaN_3_) and incubated with anti-mouse CD16/32 antibody (3 μg/mL, eBioscience, 93) for 30 min on ice. After three times washing by staining buffer, cells were further incubated with biotinylated anti-mouse OVA_254–264_ (SIINFEKL) peptide bound to H-2K^b^ (1 μg/mL, eBioscience, 25-D1.16) for 30 min on ice. After three times washing by staining buffer, cells were stained with streptavidin-PE (Sigma, St. Louis, MO, USA) and detected by a flow cytometer.

### 2.9. Mice

Seven-week-old female C57BL/6 mice (H-2^b^) were purchased from Oriental Yeast Co., Ltd. (Tokyo, Japan). All animal experiments were approved by the institutional animal experimentation committee in Osaka Prefecture University (Approval No. 19-1, approval data: 1 April, 2019) and were performed in compliance with the institutional guidelines of animal care and use.

### 2.10. Interaction of Liposomes with Splenocytes

Single cell suspension of the spleen from C57BL/6 mouse were prepared by gentle mashing and passing through a 70 μm mesh Cellstrainer™ (Falcon^®^). Erythrocytes were removed by incubating the cell pellet for 5 min in lysis buffer at 4 °C. Splenocytes were seeded into 96-well plates with 1.0 × 10^6^ cells/well and incubated with DiI-labeled liposomes (0.1 mM) in with FACS buffer (PBS containing 2% FBS) for 4 h. After washing with FACS buffer, the cells were incubated with 5 μg/mL CD16/CD32 monoclonal antibody (eBioscience) for 20 min at 4 °C to block Fc receptors, and then washed twice. The cells were stained with anti-F4/80-FITC (BioLegend, BM8), anti-CD3ε-PerCP-Cy5.5 (BD Bioscience, 145-2C11) and anti-CD11c-PE/Cy7 (BioLegend, N418) antibodies (each diluted 1:200 in FACS buffer) for 20 min at 4 °C. After washing twice, DiI-fluorescence in CD3ε^+^ cells (T lymphocytes), F4/80^+^ cells (macrophages), and CD11c^+^ cells (dendritic cells) was analyzed via a flow cytometric analysis. Cellular autofluorescence was subtracted from each data.

### 2.11. Treatment of Tumor-Bearing Mice

E.G7-OVA cells, OVA-expressing T-lymphoma, (5.0 × 10^5^ cells/mouse) were subcutaneously inoculated into the left back of C57BL/6 mice under anesthesia with isoflurane. On days 5 and 8, 100 μg of OVA-loaded liposomes were subcutaneously injected into the right backs of the mice under anesthesia with isoflurane. Tumor sizes were monitored from the day of tumor inoculation. Mice immunized with PBS were used as a control to confirm the development of tumors following the first inoculation of E.G7-OVA cells. Mice were sacrificed when tumor volumes became over 2000 mm^3^. All treated groups contained five mice.

### 2.12. Statistical Analysis

Statistically significant differences between experimental groups were determined using Prism software (v8, GraphPad). Where one-way ANOVA followed by Tukey’s HSD post hoc test was used, variance between groups was found to be similar by Brown-Forsythe test. Log-rank test was employed for analysis of survival of mice. The symbols *, **, ***, and **** indicate *P* values less than 0.05, 0.01, 0.001, and 0.0001, respectively. ns: not significant.

## 3. Results and Discussion

### 3.1. Synthesis of Mannose Residue-Introduced Carboxylated Cudlans

pH-Responsive curdlan derivatives having anchor groups (MGlu-Curd-A) were synthesized according to previous report [28] (Scheme 1). To introduce mannose residue into MGlu-Curd-A, amino group-introduced mannose derivative was prepared via thiol-ene reaction of allyl-α-D-mannopyranoside and cysteamine hydrochloride in the presence of AIBN as a catalysis [35] (Supplementary Scheme S1). Successful synthesis of 6-amino-4-thiahexyl α-D-mannopyranoside was confirmed by ^1^H NMR (Supplementary Figure S1). Amino group in 6-amino-4-thiahexyl α-D-mannopyranoside was reacted with carboxy group in MGlu-Curd-A using condensation reagent (Scheme 1). Synthesis of MGlu-Curd-A-Man was confirmed by the presence of proton signals corresponding to thioether linker (2.6–2.8 ppm) as well as curdlan main chain/mannose (3.4–4.0 ppm), MGlu units (1.9–2.3 and 0.9 ppm) and anchor units (1.2–1.6 and 0.9 ppm) (Supplementary Figure S2). The composition of polymers was calculated using peak area ratios between functional groups and showed in Supplementary Table S2. About 40% of MGlu units, 5% of anchor units, and various amounts of mannose residue (0–14%) were introduced to Curdlan. In the following section, each polymer is represented as code shown mannose residue content: Man0–14.

### 3.2. Characterization of Cudlan Derivative-Modified Liposomes

Curdlan derivative-modified liposomes were prepared by hydration of mixed thin film of EYPC and curdlan derivatives. Prepared liposome had 150–180 nm size, which corresponds to the pore size of polycarbonate membrane during extrusion (Table 1). These liposomes showed negative zeta potentials at pH 7.4, indicating the modification of carboxylated curdlan derivatives (Table 1).

**Figure 2:**
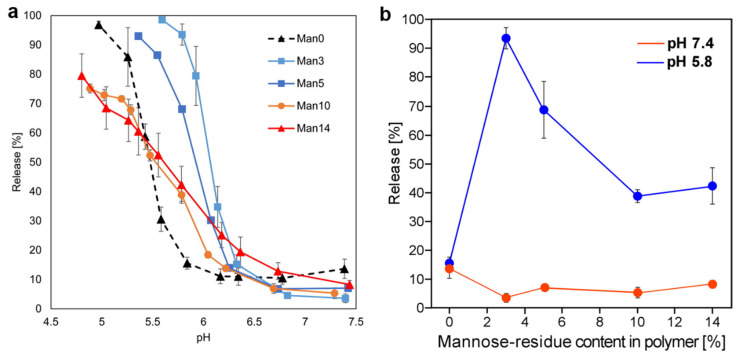
(**a**) pH-Dependence of pyranine release from curdlan derivative-modified liposomes at 30 min. (**b**) Content release from liposomes as a function of mannose-residue contents at pH 7.4 and pH 5.8.

Next, pH-sensitivity of curdlan derivatives was evaluated by detection of pyranine release from curdlan derivative-modified liposomes. Reportedly, liposomes modified with MGlu-Curd-A (Man0) showed content release below pH 6.0, indicating that MGlu-Curd-A formed hydrophobic domain after protonation of carboxy groups in the polymer and destabilized liposomal membrane via hydrophobic interaction [28] (Figure 2a). Interestingly, modification of mannose residues to curdlan derivative induced the content release at higher pH region than Man0-modified liposomes (Figure 2a). The existence of hydrophilic mannose residues in the polymer might rather promote participation of protonated MGlu units into hydrophobic parts of lipid bilayer. Especially, Man3-modified liposomes achieved >90% release of contents at pH 5.8, which corresponds to pH in the late endosome (Figure 2b). With increasing mannose residues, content release at weakly acidic region was suppressed. High introduced amounts of mannose residues might suppress efficient coil-to-globule transition of the polymers via steric hindrance, resulting in decrease of interaction with lipid membrane. Since these liposomes could induce the content release at weakly acidic pH corresponding to late endosome and lysosome (pH 5–6), these liposomes are expected to promote the content release at these intracellular organelles.

To induce endosomal escape of cargos, liposomal membrane should interact and destabilize endo/lysosomal membranes. We have already proved the membrane fusion behaviors of pH-sensitive polyglycidols and polysaccharides in weakly acidic pH using fluorescence resonance transfer [25,26]. As another evaluation, pH-sensitivity of curdlan derivative-modified liposomes was further evaluated by mixing with pyranine-loaded liposomes as a model of biological membrane (Supplementary Figure S3). At pH 7.4, pyranine release from model biological membrane was negligible irrespective with the presence of curdlan derivative-modified liposomes (Supplementary Figure S3a). This indicates that interactions between liposomes are suppressed because of a highly hydrated liposome surface by ionized carboxy groups. In contrast, curdlan derivative-modified liposomes induced pyranine release from model membrane at pH 5.5, which corresponds to late endosomal pH (Supplementary Figure S3b). These results suggest that curdlan derivatives on the liposomes can induce disruption of own liposomal membrane (Figure 2a) but also other membrane through protonation of carboxy groups. The effect of mannose residue on release profiles is almost same with the case in Figure 2: Low introduced amounts of mannose residue induced content release at higher pH region than Man0-modified liposomes, whereas high introduced amounts of mannose residue suppressed the content release compared with Man0-modified liposomes (Supplementary Figure S3c). Furthermore, release% was moderate compared with the results in Figure 2a, which reflects that the interaction between the polymer and its own liposomal membrane is easier than that between polymer-modified liposomes and other liposomes.

### 3.3. Interaction of Liposomes with Lectin

**Figure 3:**
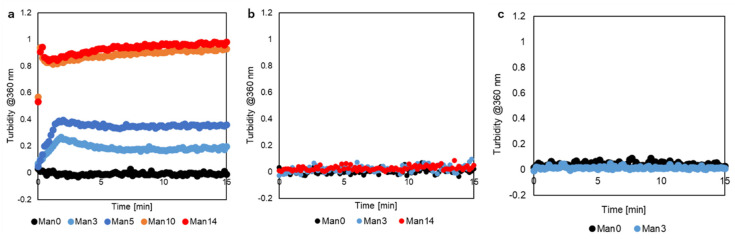
Turbidity change at 360 nm of curdlan derivative-modified liposome suspension in the presence of Con A (**a**), Con A and free mannose (**b**) and BSA (**c**).

Next, the interaction of mannose-modified curdlan derivative-coated liposomes with lectin was investigated [36]. Liposome suspension was added to Concanavalin A (Con A)-containing buffer and turbidity change of the solution was analyzed (Figure 3a). In the case of Man0-modified liposomes, no turbidity change was observed, indicating that Curdlan backbone on the liposome surface has no interaction with Con A. In contrast, mannose-modified curdlan-coated liposomes exhibited significant increase of solution turbidity, in which Con A bearing four binding sites to mannose could induce aggregation among the liposomes. Especially, turbidity of Man10- or Man14-modified liposomes suspension immediately increased after addition to Con A-containing buffer. Whereas, presence of excess mannose completely suppressed turbidity increase for Man3- or Man14-modified liposomes in Con A-containing buffer (Figure 3b). These results clearly indicate that mannose residues on the liposomes surface can be recognized by Con A specifically. Furthermore, the presence of BSA showed no effect on turbidity change for these liposomes (Figure 3c), which also supports the specific interaction of mannose residues with Con A.

### 3.4. Cellular Association of Curdlan Derivative-Modified Liposomes

**Figure 4:**
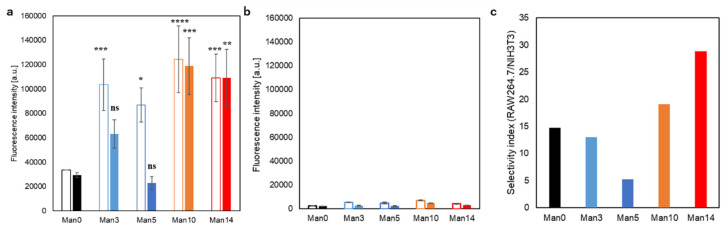
Fluorescence intensity of RAW264.7 cells (**a**) or NIH3T3 cells (**b**) treated with DiI-labeled liposomes modified with curdlan derivatives (0.1 mM) for 4 h at 37 °C in the presence (closed bars) or absence of FBS (open bars). (**c**) Fluorescence intensity ratio of RAW264.7/NIH3T3 cells. Statistical analysis was done using analysis of variance (ANOVA) with Tukey’s test. * *p* < 0.05; ** *p* < 0.01; *** *p* < 0.001; **** *p* < 0.0001 compared with Man0-modified liposomes with/without serum. ns: not significant.

Next, cellular association of curdlan derivative-modified liposomes was examined to elucidate the effect of mannose modification on APC-specific delivery performance. RAW264.7 cell, a murine macrophage cell line, was selected as a model APC. Figure 4a shows the fluorescence intensity of RAW264.7 cells treated with DiI-labeled liposomes. In a serum-free medium, mannose-modified curdlan derivative-coated liposomes exhibited 3–4 times higher cellular association than that of Man0-modified liposome, suggesting the enhancement of cellular association via mannose receptors on RAW264.7 cells. In the presence of serum, cellular association of Man3- and Man5-modified liposomes significantly decreased, whereas Man10- and Man14-modified liposomes showed almost identical cellular uptake with the cases of the absence of serum. High amounts of hydrophilic mannose residues on Man10 and Man14 might suppress the non-specific interaction with serum proteins. Cellular association to mannose receptor-negative cells was also evaluated (Figure 4b). NIH3T3 cells treated with each liposome showed quite lower fluorescence intensity than RAW264.7 cells, which reflects high phagocytic ability of RAW264.7 cells. Another explanation is the suppression of cellular association by negatively charged surface of liposomes (Table 1). In contrast, APCs including macrophages and dendritic cells have scavenger receptors to recognize anionic surface such as apoptotic cells, which also contributes high cellular association of curdlan derivative-modified liposomes on RAW264.7 cells [37,38,39]. Fluorescence intensity ratio of RAW264.7/NIH3T3 cells in the presence of serum was calculated as a selectivity index to assume the situation after subcutaneous administration of these liposomes because subcutaneous tissues are composed of fibroblasts, APCs, extracellular matrix, extracellular proteins and so on (Figure 4c). All curdlan derivative-modified liposomes showed a high selectivity index. Especially, Man10- and Man14-modified liposomes exhibited 20 and 30 values, respectively. These liposomes are expected to be taken up 20–30 times more efficiently by macrophages than by fibroblasts after subcutaneous administration.

Cellular association to primary cells was also evaluated using primary cells from spleen (Supplementary Figure S4). Single cells from spleen were incubated with DiI-labeled liposomes. Then, main cell populations in spleen were co-stained with T-lymphocyte-, macrophage- or dendritic cell-specific cell markers to distinguish cell phenotypes. As shown in Supplementary Figure S4, Curdlan derivative-modified liposomes were highly taken up by F4/80-positive macrophages and CD11c-positive dendritic cells compared with CD3-positive T lymphocytes in spleen, which is consistent with cell line results (Figure 4). Furthermore, Man14-modified liposomes showed significantly higher cellular association to macrophages and dendritic cells. These data also indicate that mannose-modified curdlan derivative-coated liposomes can promote cellular uptake by APCs in vivo.

### 3.5. Cytoplasmic Delivery of Antigen and Evaluation of Antigen Presentation

**Figure 5:**
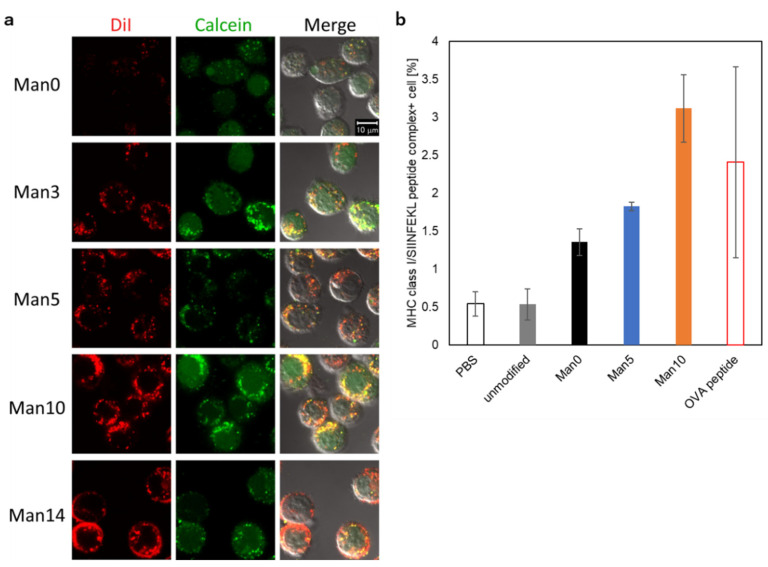
(**a**) Confocal laser scanning microscopic (CLSM) images of RAW264.7 cells treated with DiI-labeled/calcein-loaded liposomes modified with curdlan derivatives (0.1 mM) for 4 h at 37 °C in DMEM containing 10% FBS. (**b**) Expression of MHC class I/OVA epitope peptide complexes on the cells treated with OVA-loaded liposomes modified with curdlan derivatives or OVA epitope peptide (SIINFEKL).

Cross-presentation is crucial process to induce cellular immune response [13,14]. Cytoplasmic delivery of antigen or specific receptor-mediated endocytosis is regarded to be required for the induction of cross-presentation [13,14]. Therefore, cytoplasmic delivery performance of the cargo by curdlan derivative-modified liposomes was evaluated using calcein-loaded liposomes. Because calcein fluorescence is quenched when calcein concentration is high at inside of the liposomes [40], intracellular distribution of liposomal cargo can be evaluated by tracking the calcein fluorescence. Figure 5a shows confocal fluorescence images of RAW264.7 cells treated with liposomes. Red fluorescence dots were observed in most cells treated with curdlan derivative-modified liposomes, indicating that liposomes were taken up via endocytosis and located in endo/lysosomes. For the cells treated with Man10- or Man14-modified liposomes, strong DiI fluorescence was observed at the periphery of the cells, suggesting high affinity of these liposomes to macrophages and absorption onto cell surface. In most cases, calcein fluorescence was observed as both dotted and diffused states from the cells. These results indicate that these liposomes could induce both calcein release at endosomes and cytoplasmic release of calcein by destabilization of endosomal membrane responding to weakly acidic pH. These data are consistent with the data for pH-responsive content release (Figure 2) and destabilization of model membrane (Supplementary Figure S3) except for Man5-modified liposomes. Since cellular association of Man5-modified liposomes was strongly affected by serum (Figure 4a), absorption of serum proteins onto Man5-modified liposomes might suppress the interaction with endosomal membrane. We also performed the delivery of FITC-labeled OVA, model antigenic protein, using same liposomes (Supplementary Figure S5). As shown in Supplementary Figure S5, mannose modification to curdlan derivatives significantly increased the cellular uptake of both liposomes (DiI) and FITC-OVA. More importantly, FITC-OVA fluorescence was observed not only as dotted fluorescence but also diffused states within the cells (Supplementary Figure S5). These data are direct evidence for the endosomal escape of OVA molecules by mannose-modified curdlan derivatives.

Because most curdlan derivative-modified liposomes could achieve cytoplasmic content release, cross-presentation efficacy was examined using OVA-loaded liposomes (Figure 5b). RAW264.7 cells were treated with OVA-loaded liposomes and then presentation of MHC I-OVA epitope peptide (SIINFEKL) on the cell surface was detected using antibody. As a positive control, OVA epitope peptide (SIINFEKL) bound onto corresponding MHC I molecules directly was detected. As shown in Figure 5b, cells treated with PBS or normal EYPC liposomes containing OVA showed low expression of MHC I/SIINFEKL complexes. In contrast, all curdlan derivative-modified liposomes exhibited high expression of MHC I/SIINFEKL complexes. Especially, Man14-modified liposomes possessed identical performance with the case of OVA peptide-treated cells, suggesting quite high efficacy of cross-presentation. Man0- and Man14-modified liposomes might achieve efficient cross-presentation mediated by “cytosolic pathway” because of their cytoplasmic delivery performance (Figure 5a and Supplementary Figure S5), whereas Man5-modified liposomes also showed comparable cross-presentation efficacy. This might result from mannose receptor-mediated uptake, which can promote another cross-presentation route via early endosome-mediated “vacuolar pathway” [14].

### 3.6. Treatment of Tumor-Bearing Mice by Curdlan Derivative-Modified Liposomes

**Figure 6:**
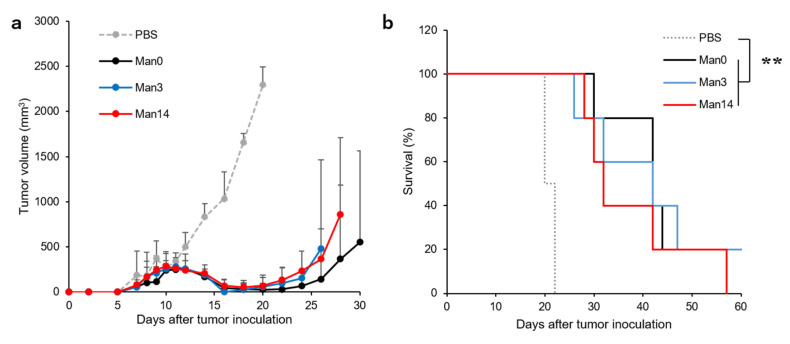
Antitumor effects induced by subcutaneous administration with OVA-loaded liposomes. C57BL/6 mice were immunized on days 5 and 8 with PBS or liposomes modified with curdlan derivatives. Changes in tumor volume (**a**) and survival (**b**) of mice were monitored after E.G7-OVA cells (0.5 × 10^6^ cells/mouse) inoculation. All treated groups contained five mice. Statistical analysis was done using Log-rank test for (**b**). ** *p* < 0.01.

Finally, in vivo performance of curdlan derivative-modified liposomes was investigated. OVA-expressing tumor cells were inoculated to mice, then OVA-loaded liposomes were subcutaneously injected to tumor-bearing mice. When PBS was injected to mice, tumor volume increased significantly and all mice reached endpoint on Day 22 (Figure 6). In contrast, subcutaneous injection of curdlan derivative-modified liposomes strongly suppressed tumor growth (Figure 6a). In most cases, tumor size became not-detectable level and grew again after Day 20 (Supplementary Figure S6). Finally, all curdlan derivative-modified liposomes significantly extended the survival of mice compared with PBS-treated group (Figure 6b). After subcutaneous injection, these liposomes might be taken up by APCs because of their high selectivity (Figure 4c). Furthermore, curdlan derivatives on the liposomes also induced maturation of APCs [28], which promotes the migration of these cells into lymph node. At lymph node, APCs presented OVA peptide onto MHC I molecules (Figure 5b) to induce OVA-specific CTLs. Activated CTLs migrated to tumor site and induced tumor cell killing. However, there is no statistical difference between liposomes with and without mannose modification. Even in Man0-modified liposomes, liposome uptake to antigen presenting cells in subcutaneous tissues might be enough because of high selectivity index (Figure 4c) and enough CTL responses might be induced for tumor regression. To see the effect of mannose modification, further high selectivity index might be required by changing the mannose density on the liposomes in addition to optimization of liposome dosage and injection timing. In all liposome-treated groups, tumor re-growth was observed (Supplementary Figure S6), which is consistent with the previous report [28]. These may result from the decrease of CTL activity after 2–3 weeks from injection, incomplete tumor cell killing due to dense extracellular matrix of tumor tissues, and induction of regulatory T cells against strong CTLs responses induced by curdlan derivative-modified liposomes. Further evaluations, such as combination with checkpoint inhibitors, cytokine gene delivery carriers, or other immunomodulatory molecules that can cancel immunosuppression in tumor tissues or can modulate tumor microenvironments, would improve the immunity-inducing performance of mannose-modified curdlan derivative-coated liposomes.

## 4. Conclusions

For this study, mannose residues were introduced to pH-sensitive curdlan derivatives to promote antigen presenting cell-specific antigen delivery performance. Mannose residue-modification promoted the content release from liposomes at weakly acidic pH. Mannose-modified curdlan derivative-coated liposomes are highly recognized by macrophage cell line and antigen presenting cells in the spleen. These liposomes also achieved the cytoplasmic delivery of cargo and cross-presentation of model antigen, which led to the induction of strong antitumor effects in tumor-bearing mice. However, mannose residue introduction did not increase in vivo performance of curdlan derivative-modified liposomes under current experimental condition, suggesting that further improvements are required, such as a combination of tumor microenvironment-modulating functions towards efficient immunity-inducing systems.

## Figures and Tables

**Table 1:** Size, PDI and ζ-potential of liposomes modified with curdlan derivatives.

Liposome	Size (nm)	PDI	ζ-Potential (mV)
Man0	144 ± 14	0.20 ± 0.03	−20.9 ± 4.0
Man3	174 ± 12	0.25 ± 0.01	−40.3 ± 0.5
Man5	157 ± 1	0.21 ± 0.02	−30.3 ± 4.0
Man10	158 ± 24	0.25 ± 0.01	−30.9 ± 0.8
Man14	181 ± 18	0.26 ± 0.01	−33.5 ± 5.2

## Supplementary Materials

The following are available online at https://www.mdpi.com/1999-4923/12/8/754/s1, Scheme S1: Synthetic route for 6-amino-4-thiahexyl α-D-mannopyranoside, Figure S1: 1H NMR chart for 6-amino-4-thiahexyl α-D-mannopyranoside, Table S1: Synthetic condition of MGlu-Curd-A-Man, Figure S2: 1H NMR charts for MGlu-Curd-A-Man, Table S2: Composition of polymers, Figure S3: Pyranine release profiles from EYPC liposomes after addition of curdlan derivative-modified liposomes, Figure S4: Cellular association of curdlan derivative-modified liposomes to immune cells in spleen, Figure S5: CLSM images of RAW264.7 cells treated with FITC-OVA-loaded liposomes, Figure S6: Individual tumor volume change for Figure 6, Table S1: Synthesis of MGlu-Curd-A-Man, Table S2: Composition of Polymers.
