# Supplementary material for: Development of Mannose-Modified Carboxylated Curdlan-Coated Liposomes for Antigen Presenting Cell Targeted Antigen Delivery
Source: Pharmaceutics. 2020 Aug 11;12(8):754. doi: 10.3390/pharmaceutics12080754 (PMC7465930; doi:10.3390/pharmaceutics12080754)
Supplement: Supplementary file 1 [file pharmaceutics-12-00754-s001.pdf]

pharmaceutics

MDPI

Supplementary Materials: Development of Mannose Residue-Introduced Curdlan-Modified Liposomes for Antigen Presenting Cell-Specific Antigen Delivery

Eiji Yuba, Yoshiki Fukaya, Shin Yanagihara, Nozomi Kasho and Atsushi Harada

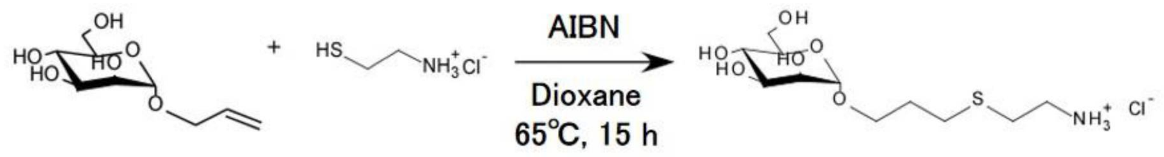

Scheme S1. Synthetic route of 6-amino-4-thiahexyl α-D-mannopyranoside.

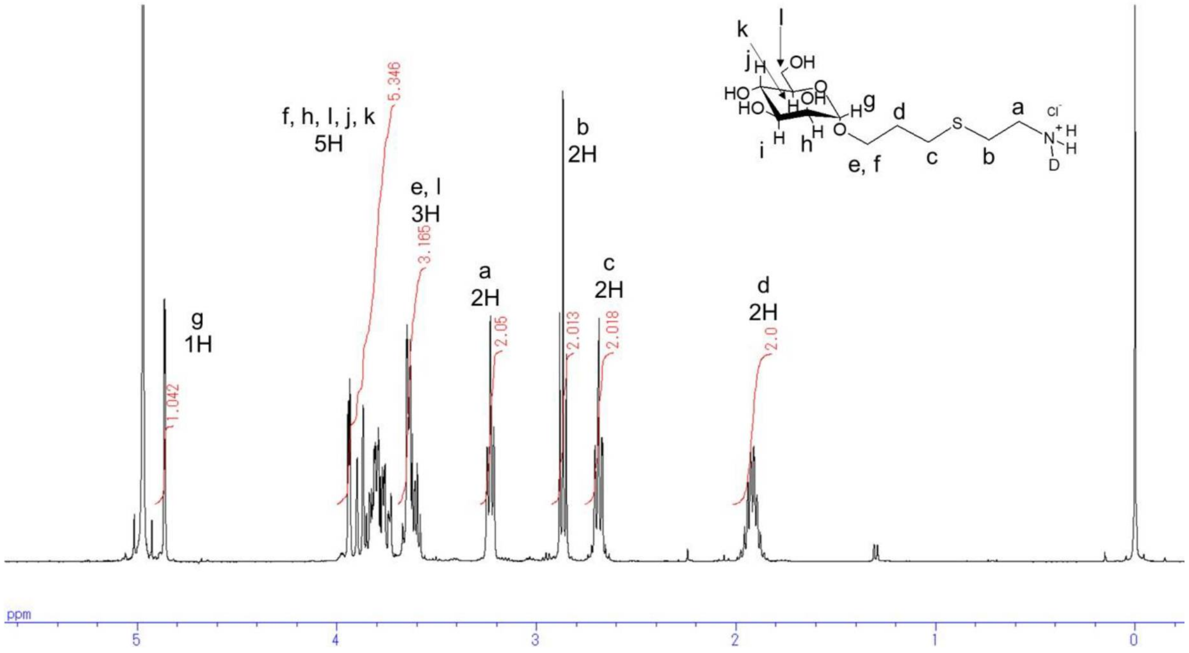

Figure S1. 1H NMR spectrum for 6-amino-4-thiahexyl α-D-mannopyranoside (D2O + DCl, 400 MHz).

Table S1. Synthesis of MGlu-Curd-A-Man.

Code	MGlu-Curd-A/mg	6-amino-4-thiahexyl α-D-mannopyranoside /mg	DMT-MM/mg	Yield /g	Yield /%	MGlu /%*	Anchor /%*	Mannose /%
**Man3**	154	59.1	51.5	129	79	31	5	3
**Man5**	153	57..6	50.9	135	82	48	5	5
**Man7**	300	59.7	49.3	294	76	49	5	7
**Man10**	154	97.3	90.1	142	72	45	6	10
**Man14**	151	130	121	179	98	55	7	14

*Determined by ^{1}H NMR.

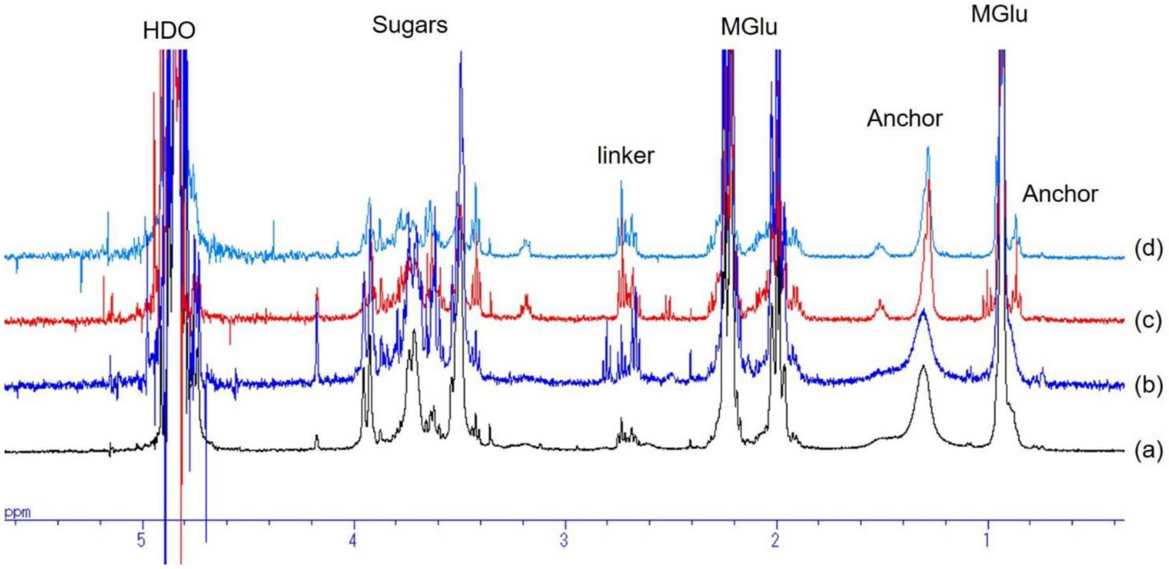

Figure S2. 1H NMR spectra for hydrolyzed MGlu-Curd-A-Man3 (a), MGlu-Curd-A-Man5 (b), MGlu-Curd-A-Man10 (c), and MGlu-Curd-A-Man14 (d) (D2O + NaOD, 400 MHz).

Table S2. Composition of Polymers.

Code	MGlu unit		Anchor unit		Mannose unit	
	/% for OH unit	/100 glucose units	/% for OH unit	/100 glucose units	/% for OH unit	/100 glucose units
**Man0**	43	129	4	12	0	0
**Man3**	31	93	5	15	3	9
**Man5**	48	144	5	15	5	15
**Man7**	49	157	5	15	7	21
**Man10**	45	135	6	18	10	30
**Man14**	55	165	7	21	14	42

*Determined by ^{1}H NMR.

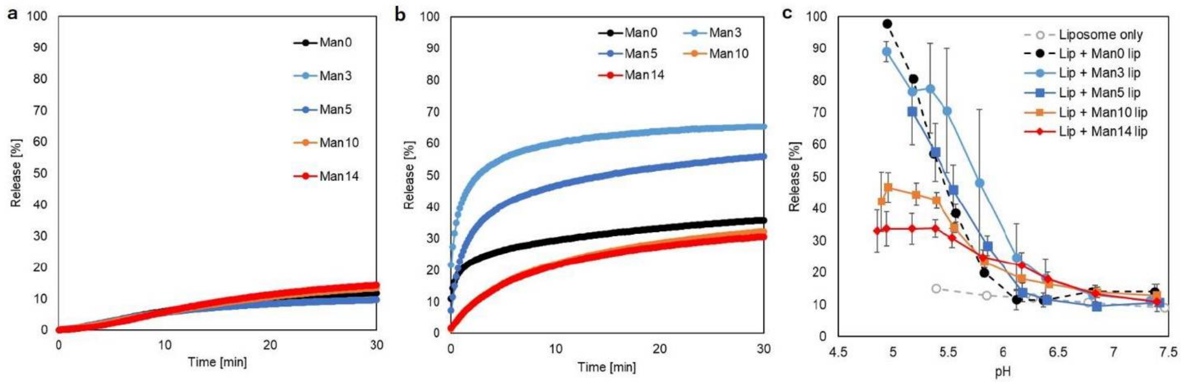

Figure S3. Time courses of pyranine release from EYPC liposome by addition of curdlan derivative-modified liposomes at pH 7.4 (a) and 5.5 (b). (c) pH-Dependence of pyranine release from EYPC liposome by addition of curdlan derivative-modified liposomes at 30 min.

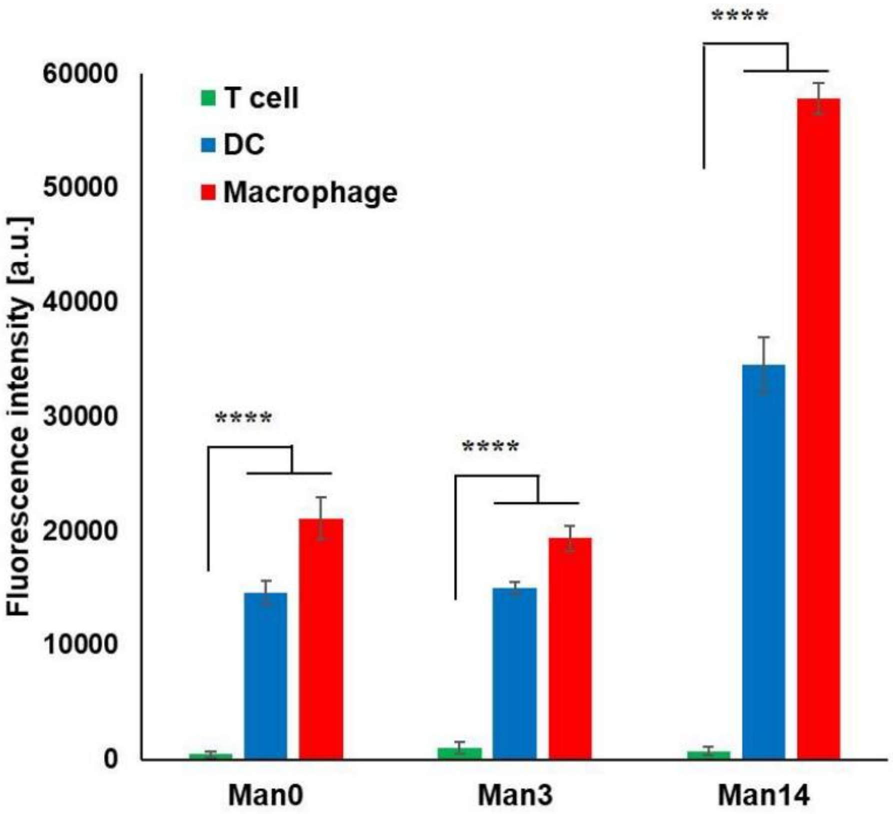

Figure S4. Mean fluorescence intensity of DiI-labeled liposome-treated splenocytes co-stained with anti-CD3 antibody (T lymphocyte), anti-CD11c+-antibody (dendritic cell), or anti-F4/80 antibody (macrophage). Statistical analysis was done using analysis of variance (ANOVA) with Tukey's test. ****$P < 0.0001$.

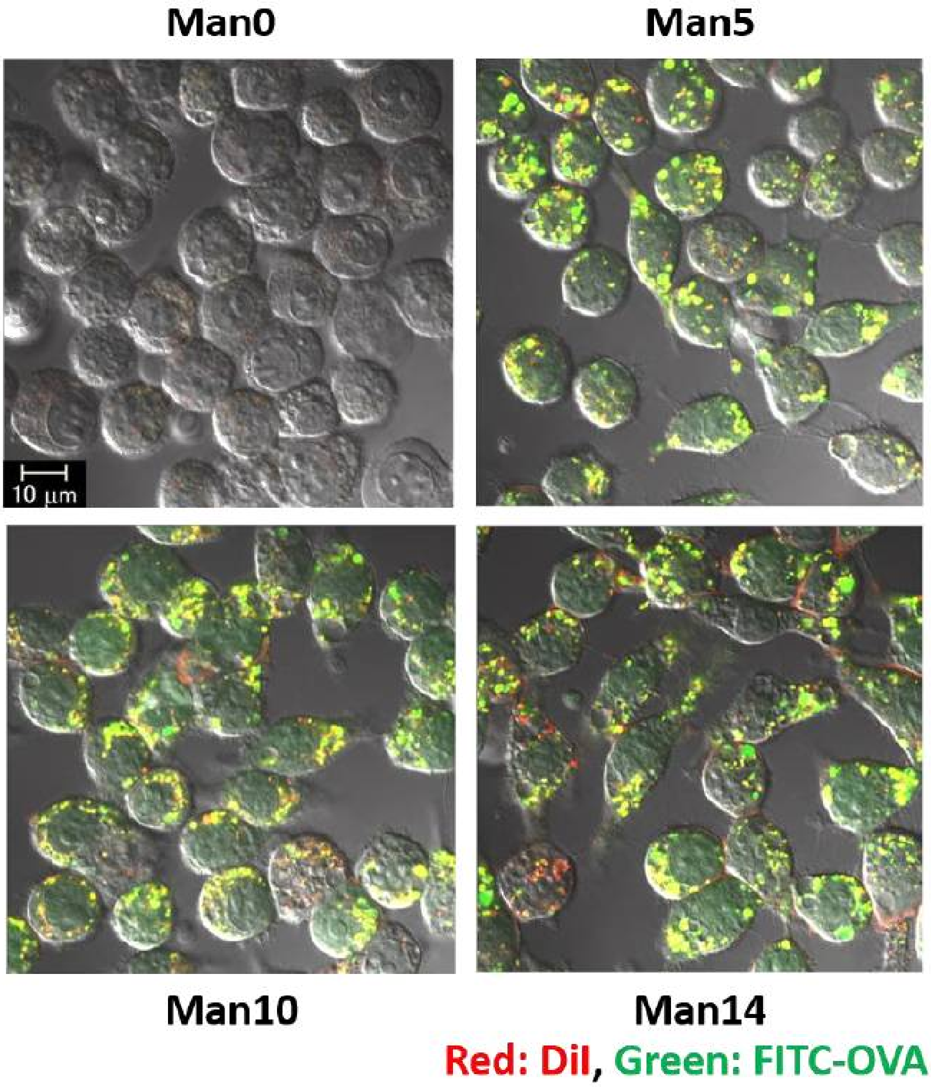

Figure S5. Confocal laser scanning microscopic (CLSM) images of RAW264.7 macrophages treated with DiI-labeled/FITC-OVA-loaded liposomes modified with curdlan derivatives (0.1 mM) for 4 h at 37 in serum-free DMEM.

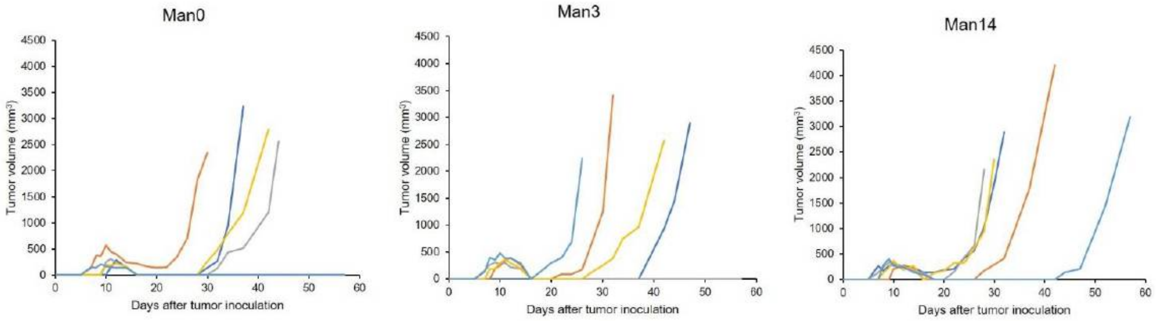

Figure S6. Individual tumor volume change of C57BL/6 mice treated with liposomes modified with various curdlan derivatives in Figure 6 (n = 5).
